# The Role of Protein S-Nitrosylation in Protein Misfolding-Associated Diseases

**DOI:** 10.3390/life11070705

**Published:** 2021-07-17

**Authors:** Yun-Jin Ju, Hye-Won Lee, Ji-Woong Choi, Min-Sik Choi

**Affiliations:** 1 Lab of Pharmacology, College of Pharmacy, Dongduk Women’s University, Seoul 02748, Korea; 20181848@dongduk.ac.kr (Y.-J.J.); 20171738@dongduk.ac.kr (H.-W.L.); 2 College of Pharmacy, Gachon University, Incheon 21936, Korea

**Keywords:** nitric oxide, protein S-nitrosylation, protein misfolding

## Abstract

Abnormal and excessive nitrosative stress contributes to neurodegenerative disease associated with the production of pathological levels of misfolded proteins. The accumulated findings strongly suggest that excessive NO production can induce and deepen these pathological processes, particularly by the S-nitrosylation of target proteins. Therefore, the relationship between S-nitrosylated proteins and the accumulation of misfolded proteins was reviewed. We particularly focused on the S-nitrosylation of E3-ubiquitin-protein ligase, parkin, and endoplasmic reticulum chaperone, PDI, which contribute to the accumulation of misfolded proteins. In addition to the target proteins being S-nitrosylated, NOS, which produces NO, and GSNOR, which inhibits S-nitrosylation, were also suggested as potential therapeutic targets for protein misfolding-associated diseases.

## 1. Introduction

Proteins responsible for important functions in cells are biosynthesized from amino acids by ribosomes, and they are tightly folded in the form of a normal tertiary structure and sometimes a quaternary structure [1]. Protein misfolding arises from abnormalities in these regulatory mechanisms. In the past, protein misfolding did not play a large role in protein research, but interest in protein misfolding began to grow significantly, as it became known as a common pathology of various neurodegenerative diseases [2]. Neurodegenerative diseases have a wide range of effects on thinking and movement, emotional recognition and memory processes. Interference with effective protein quality control is associated with neurodegenerative diseases such as Alzheimer’s disease (AD), Parkinson’s disease (PD), Huntington’s disease (HD) and amyotrophic lateral sclerosis (ALS), as well as other human diseases such as cancer, metabolic diseases and aging [3]. 

**Figure 1:**
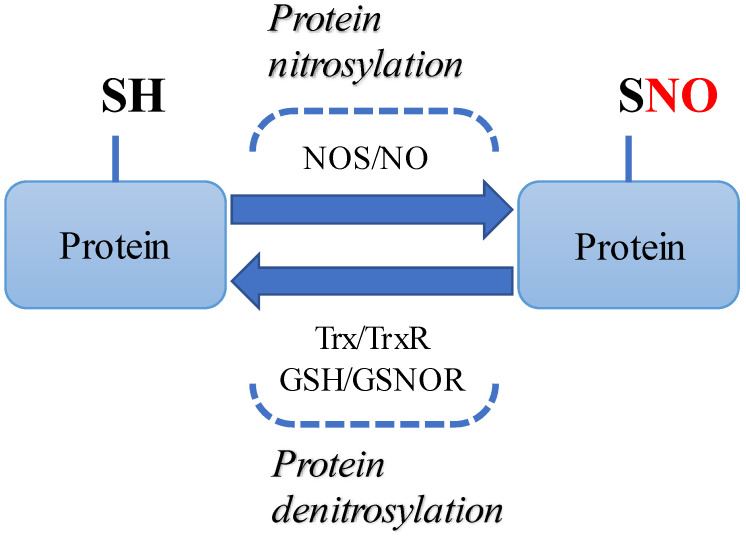
Diagram outlining the S-nitrosylation/denitrosylation pathway of proteins. During nitrosylation, nitric oxide (NO) induced by NOS binds to cysteine residues of target proteins. Conversely, during denitrosylation, thioredoxin reductase (TrxR) system and the S-nitrosoglutathione reductase (GSNOR) system remove the NO group from the S-nitrolsylated proteins.

Recently, research on nitric oxide (NO) has been actively conducted in relation to protein homeostasis, including protein misfolding [4,5]. NO is a relatively short-lived free radical, produced in many types of cells, and acts as a mediator in various intracellular signaling pathways. Excessive production of NO is harmful to the human body, which can be inactivated by antioxidant enzymes. Recently, there is increasing evidence that S-nitrosylation, which is related to the activation of NO synthase (NOS) and increase in NO, affects the process of neurodegenerative diseases (Figure 1) [6]. In addition, it is known that the activity of nitrite reductase is involved in NO generation by functional collaboration with NOS [7]. Furthermore, as seen in the cycle of NO and superoxide anion radical [8], the generation of NO is further expanded and deepened through interaction with reactive oxygen species (ROS) [9]. Collectively, NO may be involved in S-nitrosylation and directly modify biological molecules to alter the signaling response. Particularly, it has been found that NO or protein S-nitrosylation is not only involved in biological processes such as ER stress, apoptosis and autophagy, but ultimately leads to protein misfolding, mitochondrial damage, abnormal transcription factors and synaptic dysfunction [10].

Although the mechanism of protein aggregation has not been fully elucidated, here we review the latest views on chaperones, proteasomes, prions and oxidative stress involved in protein misfolding. Furthermore, the role of NO and protein S-nitrosylation related to protein misfolding will be summarized, and a target for the treatment of protein misfolding-associated diseases will be suggested.

## 2. Protein Misfolding-Related Diseases

Proteins are macromolecules that perform various functions in the human body. Mammalian cellular proteomes typically contain between 10,000 and 20,000 different proteins. To maintain the integrity of the proteome and the health of cells, protein synthesis, folding and degradation must be properly balanced and regulated. The information specifying the structure of the protein required for biological activity is encoded by the amino acid sequence of the polypeptide chain and can be prolonged up to several thousand amino acids. Proteins frequently misfold or form off-path aggregates during the folding process [11]. To avoid the misfolding of proteins and ensure proper and efficient folding, special functional proteins, including chaperones, are needed. Chaperones prevent the misfolding and aggregation of the nascent polypeptide chain during translation in the ribosome, including subsequent folding.

Protein misfolding is associated with a diverse list of diseases, which can be divided into two groups. The first is loss-of-function, which is caused by single nucleotide polymorphism (SNP) [12]. This includes cystic fibrosis, which leads to a wide range of metabolic defects. The second is toxic gain-of-function diseases [13]. Metastable proteins undergo aggregation in processes related to cytotoxicity, including the pathogenesis of neurodegenerative diseases, such as AD, PD and HD. Aggregation can be caused by inherited mutations, including HD and early-onset AD and PD [14,15]. However, despite the pathogenesis at the gene level, most cases of protein misfolding are stochastic, and appear to be age-dependently reduced in the function of protein homeostasis regulation [3].

## 3. Factors Affecting Protein Misfolding

**Figure 2:**
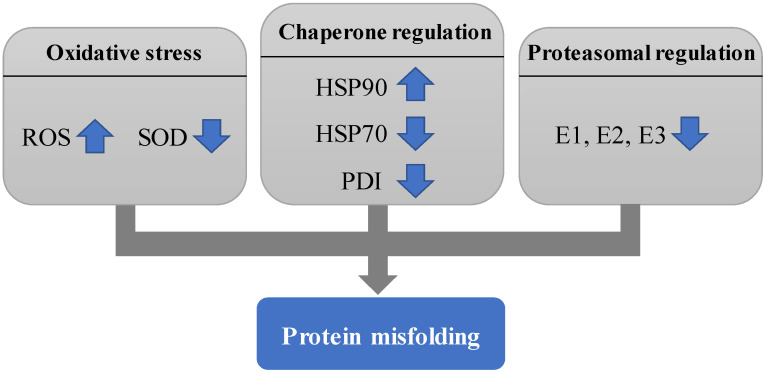
Diagram showing the factors that influence protein misfolding as described in Section 3.

First, factors that can influence protein misfolding will be reviewed. These include oxidative stress, chaperone regulation and proteasomal regulation (Figure 2).

### 3.1. Oxidative Stress

Oxygen is the most important and essential element for living organisms, but in excess it has detrimental effects. Therefore, the essential factors related to the absorption and use of oxygen are strictly controlled. Disruption of the balance between pro-oxidant and anti-oxidant systems in an organism is caused by oxidative damage to proteins, nucleic acids, lipids and carbohydrates [16]. Oxidative damage produces free radicals, including ROS, which are reactive atoms with unpaired electrons. The generated free radicals pass through the cell membrane and attack it by lipid peroxidation. It also induces structural changes and signal transduction of proteins, which further induces their aggregation. The brain is one of the most oxygen-demanding organs, and it occupies a high ratio of unsaturated fatty acids. The antioxidant system itself is weak. Furthermore, lipids are known to play a pivotal role in the brain barrier. Given the following results, it is clear that the brain is vulnerable to oxidative stress. 

When lipids are attacked by free radicals, lipid peroxidation occurs. After that, membrane fluidity decreases and membrane leakage increases [17]; this results in cleavage of membrane proteins, enzymes and receptors. Lipids in neuronal membranes are particularly susceptible to ROS, as they are very rich in polyunsaturated fatty acids. Interestingly, it has been reported that the lack of antioxidant enzymes such as superoxide dismutase (SOD) increases the phosphorylation of tau and amyloid proteins, resulting in their misfolding and aggregation [18]. 

In fact, a significant portion of the protein S-nitrosylation process, which will be discussed later in this paper, is also related to ROS formation. Therefore, the contents including the deepening of oxidation by NO or S-nitrosylation will be looked at in detail later.

### 3.2. Chaperone Regulation

Heat shock proteins (HSPs) are chaperones responsible for protein folding under normal conditions and for restoring and refolding damaged polypeptides under stress exposure [19]. Chaperones are molecular machinery that interact with a wide range of protein substrates, preventing cell damage by binding to abnormal proteins caused by heat shock and preventing their accumulation. Misfolded proteins accumulate to form insoluble plaques and fibers, a major cause of neurodegenerative diseases. Removal and refolding of misfolded proteins are mediated by HSP70, while HSP90 plays a role in increasing neurodegeneration. PD is one of neurodegenerative disorders, the main characteristic of which is loss of dopaminergic neurons in the substantia nigra [20]. A protein composed of α-synuclein and 140 amino acids is located in the entire synapse, and is genetically or neuropathologically linked to diseases. α-synuclein accumulates in intracellular filamentous inclusions as phosphorylated and ubiquitinated forms. Overexpression of HSP70 resulted in reduction of α-synuclein species in human neuroglioma cells by 50% [21]. In addition, 17-allylamino-17-demethoxygeldanamycin (17-AAG), which inhibits HSP90 and increases HSP70, reduces α-synuclein oligomerization and neurotoxicity [22]. The main hypothesis is that the cause of dementia in AD is the accumulation of misfolded proteins, amyloid β and tau. Amyloid β accumulates in both cells and neurons, and tau protein is observed both inside and outside the cell. Recently, it has been reported that amyloid β and tau synergize with each other to impair the function of neural circuits in vivo, and that this is the result of the failure of anti-amyloid β treatment [23]. Brain and animal models affected by ADs showed an increase in their communal chaperones, including HSP. In cancer clinical trials, Geldanamycin (GA), which is known to inhibit HSP90, decreased phosphorylated Tau in vivo and in vitro, suggesting that HSP90 is involved in Tau phosphorylation [24].

One of the chaperone proteins, protein disulfide isomerase (PDI), is a multifunctional protein located primarily in the endoplasmic reticulum (ER). PDI acts as an enzyme catalyzing the breakage, formation and rearrangement of disulfide bonds and as a redox regulatory chaperone interacting with the substrates [25,26,27]. So far, PDI is known to be located in various places within the cell, including the cytoplasm. Moreover, one study shows that PDI is translocated to the cytoplasm via the ER stress-induced protein reflux system [28]. PDI has been shown to be linked with diverse neurodegenerative diseases, particularly AD, and is believed to abolish neurotoxicity related with protein aggregation and ER stress [29,30,31].

### 3.3. Proteasomal Regulation

The proteasome is a key component of major proteolytic pathways. It degrades most cellular proteins in a controlled and tightly regulated manner, controlling many processes including signal transduction, gene transcription and protein quality control [32]. Proteins are tagged with a small protein called ubiquitin, and the tagging reaction is catalyzed by ubiquitin ligase. Once the protein is tagged by a small ubiquitin molecule, it signals the attachment of other ubiquitin as well. The resulting poly-ubiquitin chain binding protein can be degraded by the proteasome. During the degradation process, peptides composed of about 7 to 8 amino acids are produced, and the amino acids derived from further degradation are used to make new proteins. Malfunctions in the activity of these proteasomes can result in protein aggregation, a key process in many neurodegenerative diseases, including strokes, AD, HD and PD [33,34,35].

Accumulation of oxidized protein aggregates is higher and more noticeable in long-lived and post-mitotic cells without cell division. Since the formed aggregates do not separate into daughter cells during cell division, protein aggregation is more pronounced in post-mitotic cells. Aged neurons, for example, may have 75% of their cell volume filled with aggregates, which increases their sensitivity to apoptosis [36,37]. The consequences of aggregate formation may depend on the site and cellular location affected and the severity of formation, and metabolic disorders play an important role in protein accumulation. Post-translational modifications of proteins through metabolic and environmental factors result in changes in proteolytic susceptibility and aggregate formation. Extracellular protein deposits are degraded by phagocytosis or extracellular proteases, while intracellular aggregates are often removed by autophagy or lysosomal pathway [38,39].

Proteasome inhibitors have effective antitumor activity in cell culture and induce apoptosis. Proteasome inhibitors are the most important class of drugs that have emerged for the treatment of multiple myeloma over the past 20 years, and those are now one of the mainstays of treatment [40]. Proteasome inhibition leads to several downstream effects, including the inhibition of NF-kB signaling and accumulation of misfolded or unfolded proteins, which result in ER stress and an unfolding protein response [41].

## 4. NO and SNO in Protein Homeostasis

Up to this point, the mechanism of protein misfolding and factors affecting it were reviewed. From here, the results of studies on the effects of NO and S-nitrosylation (SNO) on protein misfolding will be summarized, and their potential as a therapeutic target will be explored.

Several neurodegenerative diseases exhibit excessive production of ROS, including superoxide anions (O2^•−^) and reactive nitrogen species (RNS), including NO [42,43]. The binding of NO groups to specific cysteine thiols of target proteins induces the formation of SNO proteins, thereby regulating protein function [44]. This biochemical process was initially characterized as a process occurring in the NMDA receptor itself. That is, NO inhibited excessive NMDA receptor activity through S-nitrosylation. It was then shown that the activity of a number of other proteins could be regulated in this way. Similar to phosphorylation, they introduced the term ‘S-nitrosylation’ to denote the biological effect of the chemical reaction of S-nitrosation. By this process, the expression, activity or transport of target proteins is affected, and may eventually mediate cytoprotective or cytotoxic effects. On the other hand, ROS, mainly superoxide anion (O2^•−^), are produced by NADPH oxidase and mitochondrial respiration [45]. The resulting superoxide anion then reacts very quickly with the free radical NO to generate the highly toxic peroxynitrate (ONOO^−^) [44].

Interestingly, it has been reported that protein misfolding can be induced by S-nitrosylation, followed by further oxidation of cysteine residues [46]. As mentioned earlier, it is known that misfolded proteins are responsible for the formation of aggregates in many neurodegenerative diseases, and consequently, can interfere with normal cellular processes or initiate apoptosis signaling pathways. Therefore, it has been demonstrated that protein S-nitrosylation can be involved in protein misfolding by at least participating in the protein peroxidation process. Considering that sporadic forms of neurodegenerative diseases are the most common, protein misfolding in these diseases may be the result of protein post-translational changes produced by oxidative or nitrosative stress [47]. That is to say, it is strongly suggested that protein S-nitrosylation may play a key role in the potential association with oxidative or nitrosative stress, which has been suggested as a pathological mechanism in all diseases related to protein misfolding.

This review focuses on specific examples demonstrating the important role of S-nitrosylation of E3-ubiquitin-protein ligase (e.g., Parkin) and endoplasmic reticulum chaperone (e.g., protein disulfide isomerase (PDI)), which contribute to the accumulation of misfolded proteins [48,49].

## 5. SNO Modification in Proteasomal Pathway

Previous studies reported that oxidative modification of the proteasome subunit inhibits protein degradation process executed by the proteasome, and this inhibition is related with aging [50]. The mechanism of this inhibition with aging, however, is still not clear. It may be elucidated with changes in the function of the proteasomal system through oxidative damages [51] or redox-dependent modifications [52]. Although the function of NO has not been fully implicated, S-nitrosylation has been shown to regulate the levels of several proteins normally controlled by proteasomal degradation. Hypoxia-inducing factor α (HIF1α), tumor suppressor protein p53 and glyceraldehyde-3-phosphate dehydrogenase (GAPDH) are included in the above [53]. Most of these results were caused by S-nitrosylation of specific substrates and chaperone enzymes, including PDI or ubiquitin E3 ligases, but they were not occurred by S-nitrosylation of the proteasome itself. It has been reported that S-nitroso-N-acetylpenicillamine (SNAP), a NO-donor, prevents the activation of the 26S proteasome by decreasing the expression of the 11S proteasome activator PA28, presumably mediated by S-nitrosylation of PA28 [54]. Based on the results of these SNAP actions, it can be said that NO may be one mechanism that regulates the response to hypoxia and the cell cycle.

## 6. S-Nitrosylation of Parkin, E3 Ligase

**Figure 3:**
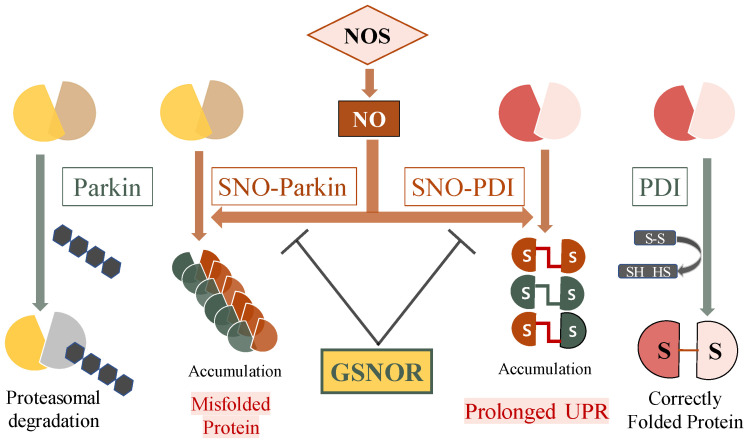
Possible reaction mechanisms for protein misfolding by aberrantly S-nitrosylated parkin (left) or PDI (right). Parkin and PDI, which are normally involved in proteasomal degradation and protein folding, respectively, are shown to induce protein misfolding after S-nitrosylation.

Parkin is a ubiquitin E3 ligase that has been associated with autosomal recessive juvenile-onset parkinsonism [55]. Some mutations in the parkin gene (PARK2) can cause familial autosomal recessive juvenile parkinsonism (AR-JP) [56] and also affect the activity of E3 ligase, which serves to route misfolded proteins into the ubiquitin-proteasome pathway for proper degradation [57]. Based on this, it seems natural that the protein substrate of Parkin E3 ligase is abnormally accumulated during neurodegeneration [58]. Unexpectedly, however, Parkin null mice do not display a PD-like phenotype in the absence of specific exogenous stress. Several possibilities have been proposed to explain these results [59,60]. As a result of the study to suggest a possible reason for the above dilemma, some groups have found that PD-related environmental toxins—including rotenone and paraquat, which induce enormous amounts of ROS and especially NO—impair the protective function of parkin for neurons through the formation of SNO-parkin [48]. Upon S-nitrosylation, the E3 ligase activity of parkin is elevated at first, but afterwards it is decreased through auto-ubiquitination or further S-nitrosylation of another cysteine thiol on parkin. In addition, increased ROS or NO levels may block E3 ligase activity by inducing further oxidation of cysteine residues in parkin (Figure 3) [61]. Once inhibition of parkin activity is mediated by S-nitrosylation, ubiquitin proteasomal degradation of misfolded proteins is initially reduced, which in turn contributes to the emergence of abnormal protein aggregates, including Lewy bodies. Moreover, there have been reports that S-nitrosylation of parkin contributes to neuronal cell death [48,62].

New experimental evidence leads us to speculate that Parkin plays an important role in the selective phagocytosis of mitochondria, and is followed by lysosomal degradation, known as mitophagy [63]. According to the findings of this study, parkin helps to guarantee mitochondria quality control by the removal of damaged mitochondria with the help of PTEN-induced kinase 1 (PINK1), which can be mutated in familial PD [64]. During this process, PINK1 increases in damaged mitochondria, and afterwards recruits parkin to mitochondria where PINK1 phosphorylates parkin and induces E3 ligase activity of parkin. Upon activation, parkin promotes a mitophagic flux by the ubiquitination of several outer membrane proteins of mitochondria. PINK1 is also known to phosphorylate Mitofusin2 and Miro1, which perform as receptors for parkin recruitment [65]. It has been reported that an initial increase in E3 ligase activity of parkin after nitrosylation promotes mitophagy, while following reduction of parkin activity interferes with mitophagy [66]. Early and transient upregulation of parkin activity could represent a negative feedback loop to protect neurons from relatively mild nitrosative stress. Conversely, late and chronic inhibition of parkin, which is caused by long-term nitrosative stress, decreases the E3 ligase activity of parkin, leading to the presence of misfolded proteins, the failure of mitochondria quality control, and possibly neuronal death. 

## 7. S-Nitrosylation of PDI, ER Molecular Chaperone Enzyme

Protein disulfide isomerase (PDI), an endoplasmic reticulum (ER) chaperone enzyme, mediates normal protein folding by thiol–disulfide exchange. Upon S-nitrosylation, the activity of PDI is suppressed. PDI is upregulated to weaken the accumulation of misfolded proteins, and following ER stress, as PDI is functioning during protein synthesis. After all, PDI is a part of the cell homeostasis system. Through S-nitrosylation, SNO-PDI compromises these ER stress responses, and subsequently increases ubiquitinated proteins, which in turn contribute to neuronal cell death in neurodegenerative diseases [67,68,69,70]. The environmentally toxic substance rotenone is known to be associated with the development of symptoms similar to PD and it contributes to the increase of SNO-PDI in a cell culture model. These results suggest that when PDI is subjected to abnormally high levels of S-nitrosylation, it leads to an intracellular increase in misfolded proteins and aggravation of ER stress. These results suggest that when PDI is subjected to an abnormally high level of S-nitrosylation, it leads to an intracellular increase of misfolded proteins, aggravation of ER stress and thereby cell death [68,70]. 

S-nitrosylation of PDI also provokes another response system, the unfolded protein response (UPR), which can result in apoptosis if the amount of abnormal proteins persist at a high level. In this regard, it has been reported that PDI itself can save the cells from proteasome dysfunction, or prolonged UPR, and not surprisingly, SNO-PDI fails to fulfill these functions (Figure 3) [49]. Wild-type SOD1 forms a homodimer, which is stabilized by a highly conserved disulfide bond. Upon S-nitrosylation, SNO-PDI becomes unable to defend cells from cytotoxicity enhanced by the aggregation of mutants or misfolded SOD1 which are observed in familial ALS. More specifically, the familial ALS-associated mutant SOD1 forms monomers with reduced disulfide bonds or insoluble multimers which have extra disulfide bonds. Hereafter, it has been reported that mutant SOD1 localizes inappropriately to the ER, and can be aggregated in intracellular inclusions, eventually contributing to apoptosis [71]. Results on the effect of changes in PDI expression—namely that overexpression of PDI reduced mutant SOD1 aggregation and inclusion formation, and that PDI knockdown increased mutant SOD1 inclusion formation—showed that PDI may function as a cytoprotective enzyme. Furthermore, as expected, S-nitrosylation of PDI causes accumulation of mutant SOD1 aggregates by reversing those cytoprotective effects [68]. After histopathological confirmation, we now know that SNO-PDI is found in the spinal cord of human patients with sporadic ALS, as well as in animal models of ALS and stroke [29].

There are other studies that have established a causal relationship between NO production and SNO-PDI formation. Mutant SOD1, through increased iNOS expression, forms SNO-PDI, followed by mutant SOD1 aggregation. Treatment with Nω-nitro-L-arginine (L-NNA), a common NOS inhibitor, alleviates these SNO-PDI-related cytotoxicities [72]. 

Taken together, these findings on SNO-PDI suggest that PDI is abnormally S-nitrosylated and loses its activity as an isomerase or a chaperone in the diseases especially dependent on NO production. Furthermore, if a new substance or mechanism that promotes specific denitrosylation of PDI is developed, it is expected that it can be used as a potential therapeutic agent to reduce ER stress and cytotoxicity related to protein misfolding.

## 8. GSNOR, a Potential Target for SNO Regulation

S-nitrosoglutathione reductase (GSNOR) is a major denitrosylase and reverts S-nitrosylation of SNO proteins to their original state, i.e., non-nitrosylated state [73]. GSNORs, which function through the denitrosylation process, have recently been reported to play a critical role in the physiological or pathological processes of target proteins. It is reported that GSNOR deletion interferes with mitochondrial dynamics and mitophagy in senescent cells through a process that promotes S-nitrosylation of Drp1 and parkin [74]. GSNOR deficiency also increases S-nitrosylation of HIF1α, which improves angiogenesis after myocardial injury [75].

Particularly, an interesting paper on the role of GSNOR involved in protein misfolding has been recently reported. When GSNOR is deleted, it enhances overall protein S-nitrosylation, accumulating protein aggregation and activating UPR [76]. This study shows that treatment with the chemical chaperone taurocholic acid (TCA) rescues the GSNOR-deleted hematopietic stem cells (HSCs), which have the regeneration defect due to 5-fluorouracil (5-FU) treatment [76,77]. These results strongly suggest a link between S-nitrosylation and following protein aggregation in HSCs in terms of blood regeneration processes.

## 9. Concluding Remarks

Significant progress has recently been made in the study of protein S-nitrosylation-related signaling pathways. As a result, we now know that the protein S-nitrosylation plays a very key role in several processes of biological function in human physiology and pathology of diseases [78,79]. NO-mediated protein S-nitrosylation affects the target proteins involved in key mechanisms, including protein folding, degradation and transport. In this paper, we reviewed recent findings regarding the positive or negative function of specific SNO proteins that are abnormally formed, such as in cytotoxic conditions or in neurodegenerative processes.

Although a comprehensive understanding of aberrant S-nitrosylation in several diseases is not yet complete, experimental results suggesting that these protein S-nitrosylation signaling pathways contribute to the mechanisms involved in the accumulation of protein misfolding are increasing. In addition, given that several aberrant SNO pathways appear only in disease-expressed tissues and not in normal tissues, this suggests that aberrant S-nitrosylation may specify the therapeutic targets for drug development. Here, key enzymes such as NOS and GSNOR, as discussed above, can be considered as preferential targets, and it is expected that the biochemical parameters of specific target SNO proteins will be referenced to discover potential therapeutic targets. Consequently, in the future, abnormal SNO proteins will be evaluated as promising molecular targets for the treatment of protein homeostasis-related diseases, including neurodegenerative diseases.

## Abbreviations

AD	Alzheimer’s disease
PD	Parkinson’s disease
ALS	amyotrophic lateral sclerosis
NO	nitric oxide
NOS	NO synthase
SNO	S-nitrosylation
RNS	reactive nitrogen species
ROS	reactive oxygen species
SOD	superoxide dismutase
HSP	heat shock protein
PDI	protein disulfide isomerase
ER	endoplasmic reticulum
HIF1α	hypoxia-inducing factor α
PINK1	PTEN-induced kinase 1
UPR	unfolded protein response
GSNOR	S-nitrosoglutathione reductase
